# Lenstar LS 900 versus Pentacam-AXL: analysis of refractive outcomes and predicted refraction

**DOI:** 10.1038/s41598-021-81146-2

**Published:** 2021-01-14

**Authors:** Henrique Aragão Arruda, Joana M. Pereira, Arminda Neves, Maria João Vieira, Joana Martins, João C. Sousa

**Affiliations:** Ophthalmology Department, Centro Hospitalar Leiria, Rua das Olhalvas, 2410-197 Leiria, Portugal

**Keywords:** Eye diseases, Applied optics, Optical techniques

## Abstract

Analysis of refractive outcomes, using biometry data collected with a new biometer (Pentacam-AXL, OCULUS, Germany) and a reference biometer (Lenstar LS 900, HAAG-STREIT AG, Switzerland), in order to assess differences in the predicted and actual refraction using different formulas. Prospective, institutional study, in which intraocular lens (IOL) calculation was performed using the Haigis, SRK/T and Hoffer Q formulas with the two systems in patients undergoing cataract surgery between November 2016 and August 2017. Four to 6 weeks after surgery, the spherical equivalent (SE) was derived from objective refraction. Mean prediction error (PE), mean absolute error (MAE) and the median absolute error (MedAE) were calculated. The percentage of eyes within ± 0.25, ± 0.50, ± 1.00, and ± 2.00 D of MAE was determined. 104 eyes from 76 patients, 35 males (46.1%), underwent uneventful phacoemulsification with IOL implantation. Mean SE after surgery was − 0.29 ± 0.46 D. Mean prediction error (PE) using the SRK/T, Haigis and Hoffer Q formulas with the Lenstar was significantly different (*p* > 0.0001) from PE calculated with the Pentacam in all three formulas. Percentage of eyes within ± 0.25 D MAE were larger with the Lenstar device, using all three formulas. The difference between the actual refractive error and the predicted refractive error is consistently lower when using Lenstar. The Pentacam-AXL user should be alert to the critical necessity of constant optimization in order to obtain optimal refractive results.

## Introduction

The Lenstar LS 900 (HAAG-STREIT AG, Switzerland) is a non-invasive biometer with OLCR (optical low coherence reflectometry), used in the calculation of intraocular lens (IOL) power through different formulas. It also provides readings on central corneal thickness (CCT), axial length (AL), anterior chamber depth (ACD), keratometry, pupillary diameter, retinal thickness measurements and white-to-white distance^1^.

The Pentacam (OCULUS, Germany) uses a rotating Scheimpflug camera which provides a three-dimensional scan of the anterior segment of the eye. The more recent Pentacam-AXL (OCULUS, Germany), by means of using partial coherence interferometry technology to obtain measurements of axial length (AL), has gained the ability to perform non-contact biometry. With this new feature, and in combination with the measurements obtained with the Scheimpflug rotating camera, this device can now perform the calculation of intraocular lens (IOL) power required in cataract and refractive surgery. Accurate and reliable measurements of ocular parameters and consequently of IOL power are essential to the success of surgery and patient satisfaction^2–5^.

We previously compared measurements of axial length (AL), anterior chamber depth (ACD) from the corneal epithelium (ACD ext) and endothelium (ACD int) to the anterior surface of the lens, central corneal thickness (CCT) and keratometry readings of the steepest and flattest corneal meridians (K1 and K2) obtained with both devices. Mean values of K1, K2 and CCT were different. Keratometry readings performed with the Lenstar were higher than with the Pentacam. CCT measurements retrieved from the Lenstar were also higher compared to the Pentacam. AL and ACD readings were not significantly different. We also performed IOL calculation with the Lenstar and Pentacam with SRK/T, Haigis and HofferQ formula. The comparison of these variables showed significant differences IOL power in all formulas between both devices, with the Pentacam showing higher IOL power values with all the three formulas. A possible reason for the differences found in IOL power calculation might be due to the different keratometric readings between both devices^6^.

Our goal now is to compare refractive outcomes after cataract surgery, evaluating the differences between real refraction and the predicted refraction obtained with both the Lenstar and the Pentacam.

## Methods

This retrospective sub-analysis to the previous study^6^ included 136 eyes of 79 patients undergoing cataract surgery between November 2016 and August 2017 at Leiria Hospital Centre. Informed consent was obtained for the collection of data that is part of our standard practice and adhered to the tenets of the Declaration of Helsinki. The study protocol was approved by the institution’s review board.

Exclusion criteria included patients with previous anterior segment or vitreoretinal surgery, white mature or traumatic cataracts, patients with central corneal opacities or high corneal astigmatism and a difference in measurements of axial length bigger than 0.3 mm between both eyes. This latter exclusion criteria is due to the possibility of measurement error that is raised by the Pentacam´s software, suggesting the user performs a confirmatory AL measurement with another device or with ultrasound immersion biometry.

Biometry with the Lenstar LS900 (HAAG-STREIT AG, Switzerland) and Pentacam (Pentacam-AXL, OCULUS, Germany) was performed. Parameters retrieved were AL, IOL power chosen for each surgery and predicted refraction using Haigis, SRK/T and HofferQ with the selected IOL with both devices. IOL selection for surgery was performed with biometry data from our reference biometer, the Lenstar LS900, using the formula that yielded a value of diopter power closest to emmetropization.

Uncomplicated phacoemulsification with IOL implantation in the capsular bag was performed on 104 eyes by the same surgeon. IOL type was Acrysoft IQ monofocal SN60WF in all surgeries. Main incisions were done on the steepest corneal axis with a 2.4 mm keratome in all cases.

Post-operatively, eyes with IOL not in-the bag or with a secondary procedure occurring at the same time as phacoemulsification were excluded from analysis. Four to six weeks after surgery, the spherical equivalent (SE) was derived from the objective refraction obtained with auto refractometer (NIDEK ARK-1a). Mean prediction error (PE) or the difference between the actual refractive error and the predicted refractive error was calculated, based on the predicted spherical equivalent derived from both the Lenstar and the Pentacam separately, for the IOL power selected for the surgery, with the SRK/T, Haigis and HofferQ formulas. Mean absolute error (MAE) and the median absolute error (MedAE) were also calculated for better characterization and comparison purposes. The percentage of eyes within ± 0.25, ± 0.50, ± 1.00, and ± 2.00 D of MAE was also determined in order to compare refractive predictability.

### Statistical analysis

The data was analyzed with SPSS version 22 software (IBM Inc., Chicago, Illinois, USA). Normality was guaranteed by the Kolmogorov–Smirnov test and graphical analysis. Statistical analysis was performed using descriptive statistics and AL was compared using a paired Student t-test. The Friedman test was used to test the difference among instruments for PE, MAE and MedAE. The tests were considered significant when *p* < 0.05 with 95% confidence intervals.

### Ethical approval

All procedures performed in studies involving human participants were in accordance with the ethical standards of the institutional and/or national research committee and with the 1964 Helsinki declaration and its later amendments or comparable ethical standards.

### Informed consent

Informed consent for anonymous biometric data collection was obtained from all individual participants included in the study, as part of the departments standard of care practices.

## Results

Our study sample included 136 eyes of 79 patients, 53.2% were female. Mean age was 76.2 ± 6.8 years. From the initial sample, 104 eyes from 76 patients, 35 males (46.1%) and 42 females (55.3%), underwent uncomplicated phacoemulsification with IOL implantation in the capsular bag.

Mean AL measured with the Lenstar was 23.01 ± 0.77 mm [20.89; 24.86] and 22.97 ± 0.77 [20.84; 24.78] mm with the Pentacam. There were no significant differences between both variables (*p* < 0.0001). Mean SE 4–6 weeks after surgery was − 0.29 ± 0.46 D. Evaluating the difference between predicted refraction and actual refraction using the SRK/T formula, it was found that the final refractive outcome was 0.07 diopters (D) more hypermetropic than the predicted refraction when using the Lenstar and 0.16 D more myopic when using the Pentacam (*p* = 0.001). In a similar way, using the Haigis formula, the final refractive outcome was 0.09 D more hypermetropic than predicted refraction using Lenstar and 0.23 D more myopic when using the Pentacam (*p* < 0.0001). When using the HofferQ formula, it was observed that final refractive outcome was 0.04 D more hypermetropic using the Lenstar and 0.25 more myopic using the Pentacam (*p* < 0.0001). The MAE did not differ significantly between devices (*p* = 0.2 SRK/T; *p* = 0.21 Haigis; *p* = 0.31 HofferQ). PE, MAE and MedAE are further described in Table 1.

**Table 1:** Postoperative outcomes: Refraction prediction error with the Lenstar and Pentacam.

	SRK/T Lenstar/Pentacam		Haigis Lenstar/Pentacam		HofferQ Lenstar/Pentacam	
PE (Mean + SD) (D)	0.07 ± 0.48	− 0.16 ± 0.5	0.09 ± 0.47	− 0.23 ± 0.49	0.04 ± 0.48	− 0.25 ± 0.47
Range (D)	− 0.96 to 1.73	− 1.62 to 1.77	− 0.91 to 1.76	− 1.59 to 1.06	− 0.93 to 1.67	− 1.25 to 1.0
MAE (Mean + SD) (D)	0.37 ± 0.32	0.40 ± 0.33	0.36 ± 0.31	0.42 ± 0.34	0.36 ± 0.31	0.43 ± 0.32
MedAE (D)	0.29	0.28	0.28	0.35	0.29	0.41

*PE* mean predicted error calculated as the difference between the measured and predicted postoperative refractive spherical equivalent, *MAE* mean absolute error, *MedAE* median absolute error.

Percentage of eyes within ± 0.25, ± 0.50, ± 1.00 and ± 2.00 D MAE using SRK/T with the Lenstar device was 45.2%; 26.0%; 19.2% and 5.8% respectively. Using the same formula in the Pentacam, the percentage of eyes within ± 0.25, ± 0.50, ± 1.00, and ± 2.00 would be 40.4%, 31.7%, 23.1% and 4.8%. If the refractive outcome was considered as the proportion of those who differed from the predicted refraction then 74% were within 0.5 D and 94% were within 1.0 D when the Lenstar was used, compared with 72% within 0.5 D and 95.2% when using the Pentacam with the SRK/T formula.

**Figure 1:**
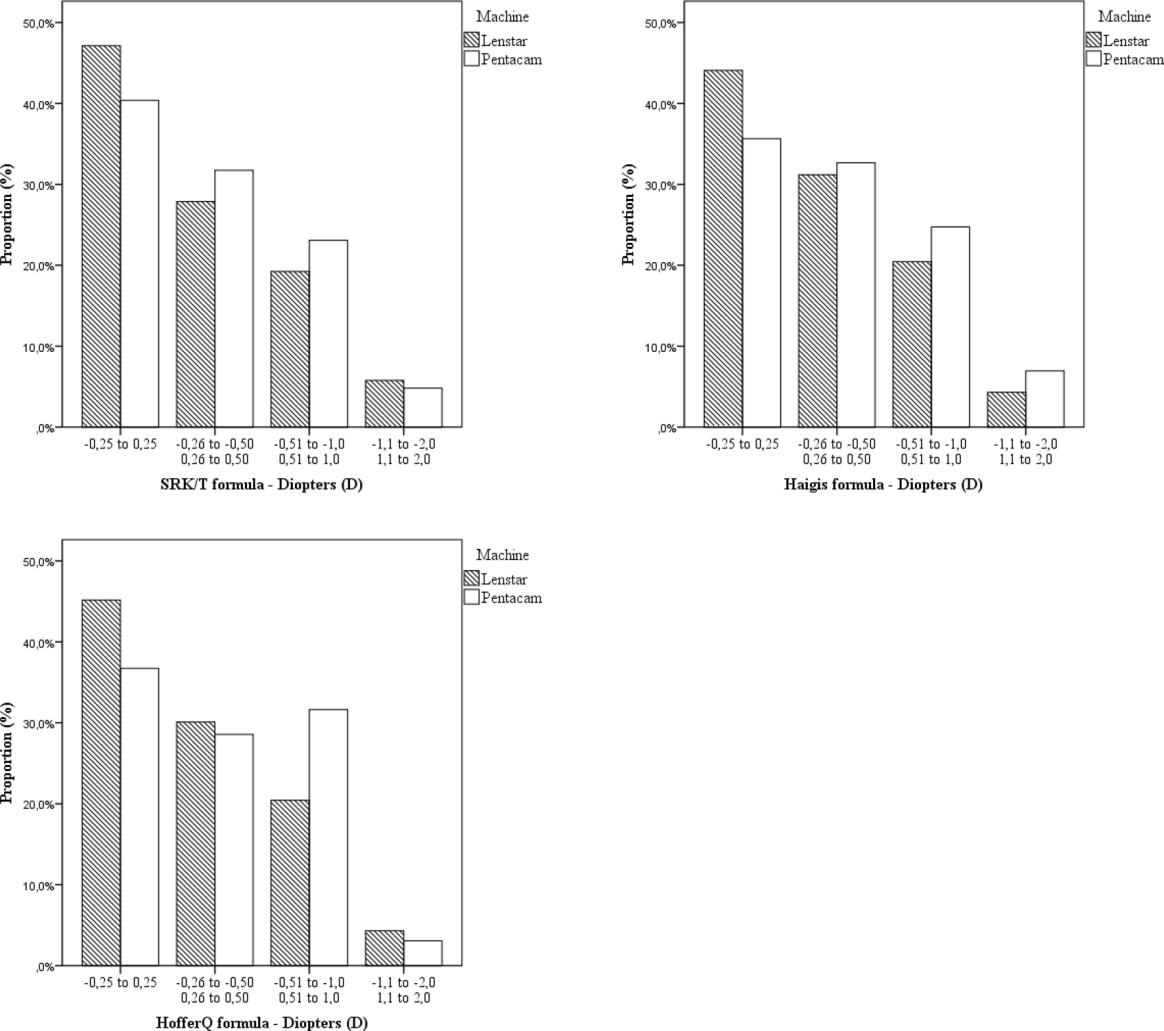
Proportion of eyes whose postoperative refraction differed from preoperative predicted refraction as determined by the Lenstar and the Pentacam with SRK/T, Haigis and Hoffer Q formulas.

Proportion of eyes whose postoperative refraction differed from preoperative predicted refraction with the SRK/T, Haigis and Hoffer Q formulas using both devices, is illustrated in Fig. 1.

## Discussion

The precise acquisition of ocular parameters is the first step to correctly determine the IOL power required to achieve target refraction as predictability of the refractive error after cataract surgery in biometry is key to meet high standards of refractive outcomes.

In our previous study^6^, AL and ACD readings showed excellent correlation and agreement but keratometry readings performed with the Lenstar were significantly higher than with the Pentacam. Moreover, IOL power closest to emmetropization calculated with the Pentacam showed higher values for the same eye.

Muzyka-Woźniak et al.^7^ found flatter corneal curvature measurements in the Pentacam-AXL when comparing it with the IOL Master 500. ACD measurements were not statistically significant and differences in AL were clinically insignificant. They showed the proneness of the Pentacam-AXL to provide higher calculated IOL power. They suggested it could be explained by its flatter keratometric measurements and proposed optimization of constant used in IOL power calculation formulas. These results are in line with our previous work^6^. Sel et al.^8^ have reached similar conclusions when comparing IOL Master to Pentacam-AXL. In contrast, Shajari and colleagues^9^ found no significant differences in keratometric parameters.

In this study, analyzing the actual refractive outcomes, we found that the difference between the actual refractive error and the predicted refractive error is consistently lower when using Lenstar. This is represented by a lower value of mean error and mean absolute error when using the values ​​obtained with Lenstar. Percentage of eyes within ± 0.25 D MAE were also larger with the Lenstar device, using all three formulas. When using the SRK/T formula the difference in MAE between both machines was inferior thus implying greater inter-device proximity when using this formula.

Refractive outcomes using optic biometry and other biometry techniques such as applanation and immersion were compared by different authors^10–14^.

Naicker et al.^10^ found no significant differences in the predicted post-operative refractive outcome between the Lenstar LS900 and the Immersion A-scan ultrasound devices. Trivedi et al.^11^ showed Immersion A-scan biometry would be more desirable for IOL power calculation on a pediatric population. Landers^12^ and colleagues compared the IOL Master to immersion ultrasound and revealed a more predictable refractive outcome with the IOL Master, with patients’ SE closer to target refraction. Lam et al.^13^ found the Lenstar superior to the immersion biometry in refractive outcomes. Nemeth et al.^14^ showed slightly better outcomes with optical biometry comparing to immersion biometry with no optimized formulas and significantly better results with optimization of IOL-constants.

Refractive outcomes comparison between an optical biometer and an instrument with Dual Scheimpflug/Placido’s discs technology and low coherence interferometry biometry (Galilei G6 ZIEMER OPHTHALMIC SYSTEMS AG) have been performed^15,16^. IOL powers determined by the IOLMaster (CARL ZEISS MEDITEC), were compared to the refractive outcome that would have been achieved using IOL powers determined with the Galilei G6 Lens Professional, a Dual-Scheimpflug Corneal Topographer/Keratometer (ZIEMER OPHTHALMIC SYSTEMS)^15^. No significant differences were found between them. Jung et al.^16^ comparing the IOL Master and the Galilei G6 found that the proportion of eyes with an MAE within 0.5 D was 85.0% for the IOLMaster and was 80.0% for the Galilei G6 based on the SRK/T formula.

The Pentacam-AXL accounts for astigmatism of both anterior and posterior corneal surfaces. It estimates changes in the corneal shape suffered by previous refractive surgery (PRK or LASIK). These functions come as highly desirable in patients with previous refractive surgery. Patients with these characteristics represent a challenge in IOL calculation, because, among other reasons, the keratometric index of 1.3375 used to convert the anterior corneal curvature into diopters is no longer valid after LASIK or PRK^17^. For these reasons, this device represents a resource of great value in achieving the best refractive results in populations with characteristics as diverse as those we find today.

The Acrysoft IQ monofocal SN60WF constants for the Pentacam-AXL were the same as for the Lenstar in our study. In the same way as Muzyka-Woźniak et al.^7^, in the comparison of the Pentacam with the IOL Master, we believe these constants are not the ideal for the Pentacam-AXL and are the main reason for IOL calculation and mean error dissimilarity with the Lenstar. However, some studies with constant optimization have shown diverging results in the incidence of prediction errors in IOL calculation with the Pentacam^18,19^.

The main limitations to this study are the inclusion of both eyes, the lack of constant optimization to the formulas and the execution of biometry exams by several technicians. Regardless, we believe that this comparison is relevant to the daily clinical practice as it alerts the Pentacam-AXL user to the critical necessity of constant optimization in order to obtain optimal refractive results.
